# World Malaria Day — April 25, 2014

**Published:** 2014-04-25

World Malaria Day is commemorated on April 25, the date in 2000 when 44 African leaders met in Abuja, Nigeria, and committed their countries to reducing malaria-related deaths. Between 2000 and 2012, the scale-up of effective malaria prevention and control interventions saved more than 3.3 million lives and decreased malaria mortality by 45% globally and 49% in sub-Saharan Africa. In spite of those accomplishments, an estimated 207 million cases of malaria occurred globally in 2012, resulting in an estimated 627,000 deaths. Because of increases in insecticide and drug resistance and changes in malaria epidemiology as a result of scaled-up interventions, new approaches are needed to sustain progress in malaria control and lead toward elimination. World Malaria Day 2014’s theme, “Invest in the Future: Defeat Malaria,” is a reminder of the challenge and the ultimate goal.

CDC supports global malaria control efforts through the President’s Malaria Initiative, a U.S. government interagency initiative to reduce malaria incidence and mortality in 19 countries in sub-Saharan Africa and in the Greater Mekong Subregion in Asia. This effort has helped deliver millions of insecticide-treated mosquito nets, antimalarial drugs, and rapid diagnostic test kits to ensure that persons at risk for malaria will have access to life-saving prevention and treatment. CDC also conducts multidisciplinary strategic and applied research globally to increase knowledge about malaria and develop safe, effective interventions that can lead to the elimination and eventual eradication of malaria. Additional information regarding CDC’s malaria activities is available at http://www.cdc.gov/malaria.
